# Systemic approach to green infrastructure and nature-based solutions uptake: Insights from the Polish cities

**DOI:** 10.1007/s13280-025-02336-0

**Published:** 2026-03-24

**Authors:** Iwona Zwierzchowska, Małgorzata Stępniewska, Grzegorz Wolszczak

**Affiliations:** 1 Department of Integrated Geography, Faculty of Human Geography and Planning, Adam Mickiewicz University in Poznań, ul. B. Krygowskiego 10, 61-680 Poznan, Poland; 2 Department of Urban, Land, Disaster Risk Management, World Bank, Vienna, Austria

**Keywords:** Municipal practices, NBS, Poland, Practitioners' perspective, Urban challenges

## Abstract

Cities face many environmental, social, and economic challenges. Green infrastructure (GI) and nature-based solutions (NBS) are the tools that contribute to tackling them and improving the quality of life of urban citizens. To strengthen the competencies of cities in achieving sustainable development, the Polish Ministry of Development Funds and Regional Policy led, in collaboration with the World Bank, a flagship project of the National Urban Policy 2030—the Cities' Partnership Initiative (CPI) 2021–2023. Within CPI, ten Polish cities participated in the Green Network Group. Based on Green Network's work results, we aim to: 1. Reveal contemporary key urban challenges that the GI and NBS can address; 2. Indicate barriers in the planning, design, execution, and maintenance of GI or NBS that hinder their systemic development (legal, organizational, substantive, financial); 3. Propose improvements to the GI and NBS systemic development from the perspective of practitioners reperesenting cities. The findings result from a series of workshops and Municipal Action Plans developed by participants to address city-specific challenges. The results showed that urban challenges are multidimensional, and GI and NBS are recognized as multi-beneficial solutions. Municipalities struggle with legal, organizational, substantial, and financial shortages, but at the same time, they indicate various improvements that are needed to strengthen the systemic approach to GI and NBS uptake.

**Supplementary Information:**

The online version contains supplementary material available at 10.1007/s13280-025-02336-0.

## Introduction

The global phenomenon of urban development is manifested, on the one hand, in the densification of the built-up areas and the displacement of greenery from cities (Grêt-Regamey et al. 2020), and on the other hand, in the expansion of development areas into previously undeveloped spaces in the suburban zone (EEA 2006; Behnisch et al. 2022). The consequences of increasing intensity of built-up and progressing soil sealing impact biodiversity (Li et al. 2022), water regimes (McPhearson 1974), climate conditions (Oke et al. 2017), and availability of and access to green recreation areas (Puplampu and Boafo 2021), among others. Combined with the effects of climate change, manifested by more frequent heavy rains, but also periods of drought and unfavorable thermal conditions in summer, cities are facing more frequent phenomena such as flash floods or flooding in urban areas (Pińskwar et al. 2023), the urban heat island, the occurrence of hot days and very warm or tropical nights (Półrolniczak et al. 2018). All of the above calls for solutions that address multiple challenges to enhance cities' resilience to climate change and improve residents' well-being (Grêt-Regamey et al. 2020).

The two concepts gaining attention in practice, addressing those and other challenges, are green infrastructure (GI) and nature-based solutions (NBS) (Hansen et al. 2023). Earlier embedded in practice, GI—understood as a strategically planned network of green areas designed and managed to provide a wide range of ecosystem services (EC 2013)—prioritizes connectivity across multifunctional ecosystems as a key feature in planning (Langemeyer and Baró 2021). NBS were introduced later by the EU (2015) as *“actions inspired by, supported by, or copied from nature,”* designed to effectively and adaptively address societal challenges while simultaneously contributing to human well-being and biodiversity benefits (Cohen-Shacham et al. 2016). Building on this concept, UNEA 5.2 (2022) further defines these actions as oriented toward the protection, conservation, restoration, sustainable use, and management of natural or modified terrestrial, freshwater, coastal, and marine ecosystems, thereby addressing social, economic, and environmental challenges while enhancing human well-being, ecosystem services, resilience, and biodiversity benefits. Therefore, NBS that are targeted interventions addressing specific societal challenges can be seen as localized nodes within broader GI networks, which contribute to ecosystem service flows within GI, aligning a multi-scale planning approach (Langemeyer and Baró 2021). However, it should be noted that the two concepts of GI and NBS are defined differently across various contexts, often overlap and interpenetrate, reflecting their conceptual and practical interconnectedness. As an umbrella concept, NBS cover a range of ecosystem-based approaches, including restorative, issue-specific, infrastructure, management, and protection approaches (Cohen-Shacham et al. 2019) and encompasses various solutions. Taking into account the level of human interference applied to biodiversity and ecosystems and the level of enhancement of ecosystem services, NBS are grouped into three main types of actions, including (1) no or minimal intervention to improve ES, (2) management approaches that develop sustainable and multifunctional ecosystems to improve ES, (3) intensive management or creation of new ecosystems (Eggermont et al. 2015). Those types relate to better use of existing natural or protected ecosystems, improved management of managed and degraded ecosystems, and the creation of new ecosystems (Cohen-Shacham et al. 2016). Following this approach, Nikolaidis et al. (2019) proposed more detailed (sub) categories for each type of NBS. Similarly, the creation of new GI elements, enlarging existing ones, enhancing their quality or multifunctionality, and various usage and actions promoting GI usage can be recognized at the municipal level as NBS-related activities in urban policy documents (Zwierzchowska et al. 2019). A valuable perspective on NBS is also provided by assessing their co-benefits and impact in relation to the specific challenges they address (Raymond et al. 2017). NBS can also be classified based on the dominant medium (land, water, built-up areas), spatial scale (micro, meso, and macro levels), and primary function (Castaldo et al. 2025).

### From theoretical concept to practical perspective on urban NBS implementation

The EU is promoting wider use of GI and NBS in cities through policies such as the 2030 Biodiversity Strategy, which requires cities with over 20 000 inhabitants to develop Urban Greening Plans (EC 2020). Guidance on Urban Nature Plans has been issued to support local authorities in this process (EC 2023), and the Ecosystem Restoration Law further mandates no net loss—and ultimately an increase—of national urban green space and tree canopy cover (EP 2024).

Although GI and NBS offer numerous benefits (IUCN 2020), the transition from theoretical concepts to practical implementation remains challenging (Davis et al. 2024). The implementation of NBS in cities is typically linked to urban planning and policy processes (Raymond et al. 2017), which take place in the wider dynamically changing context (e.g., legal systems, organizational structures, economic conditions, knowledge, and the natural environment) that affects the development of practices and uptake of new approaches. As a result, these approaches are still not widely adopted as mainstream practices (Dorst et al. 2022).

Given that urban municipalities play a key role in the GI and NBS implementation process, there is a need to strengthen practice-oriented studies that advance knowledge relevant to capacity building and governance of NBS at the local level (Wickenberg et al. 2021). Researchers only started to reflect on city representatives' experiences and points of view in this area (e.g., Wihlborg et al. 2019; Frantzeskaki et al. 2020; Toxopeus and Polzin 2021; Dorst et al. 2022; Duffaut et al. 2022; Adams et al. 2024; Mercado et al. 2024; Tran et al. 2024).

In Poland, cities are engaging in NBS- and GI-related initiatives through involvement in EU-funded projects, e.g., Wrocław (pocket parks), Kraków Metropolis (fund finder for blue-green infrastructure projects), Katowice (NBS for pollution reduction and regenerative development), Gdańsk (rain gardens), Poznań (nature-oriented playgrounds), Warsaw (blue-green-gray infrastructure), Łódź (linear park) (CORDIS 2025). Nevertheless, such participation has so far been mainly limited to a few larger urban centers. Consequently, NBS implementation in Poland is still in its early stages, with local authorities tending to focus on individual solutions rather than adopting a comprehensive, systemic approach (Pancewicz et al. 2023). At the same time, NBS are more commonly introduced in cities with higher per capita budget revenues, while their presence remains marginal in rural municipalities (Małecka-Ziębińska and Janicka 2022). This pattern reflects broader trends at the EU level, where the majority of NBS projects are concentrated in urban ecosystems (Hudson et al. 2023).

Although the concepts of GI and NBS penetrate the practice of decision-making in polish cities—they are not widely recognized, and their uptake remains uneven, ambivalent, selective, and somewhat superficial (Baravikova 2020). This lack of recognition can be linked to the absence of GI or NBS definitions in legal documents (Legutko-Kobus et al. 2024). On the other hand, municipal authorities have implemented NBS-like measures without necessarily being aware of the appropriate terminology (Małecka-Ziębińska and Janicka 2022), and similar solutions were already in use even during the socialist period (Kronenberg et al. 2017).

To move beyond theoretical discussions on the systemic integration of GI and NBS into planning, design, and management—and to contribute to studies focused on practitioners' perspectives—we based our study on the experiences of representatives of 10 cities participating in the Green Network Group within the Cities' Partnership Initiative (CPI) 2021–2023. This approach responds to the broader need for practice-oriented knowledge that our study seeks to address. In this paper, we aim to:

1. Reveal contemporary key urban challenges that the GI and NBS can address.
2. Indicate barriers in the planning, design, execution, and maintenance of GI or NBS that hinder their systemic development (legal, organizational, substantive, financial).
3. Propose improvements to the GI and NBS systemic development from the perspective of practitioners representing cities.

In this study, the mentioned issues are examined at the municipal level from the perspective of municipal practitioners.

### Case study description

The Cities’ Partnership Initiative (CPI) is a strategic project of the Polish Ministry of Development Funds and Regional Policy (MDFRP) led in collaboration with the World Bank (WB) (WB 2023; MDFRP^1^). It aims to strengthen the competencies of cities and central administration in selected thematic areas (so-called networks) related to the sustainable development of cities and enhance networking among the project participants. The Green Network was one of three CPI 2021–2023 thematic networks (alongside the Digital and PPP Initiatives Networks) focusing on green urban investments (WB 2023).

The substantive work of the Green Network was framed and led by WB experts in collaboration with the MDFRP and with support from the Ministry of Climate and Environment (WB 2023). Three main thematic streams that were defined included GI planning, environmental aspects of urban regeneration and rehabilitation, and adaptation to climate change.

Within these main thematic streams, cities engaged in the CPI Green Network worked on individual urban challenges relevant to their specific contexts. The Network's work was focused on:

1. Preparation of a Municipal Action Plan (MAP) that describes each city’s solution to its specific sustainable development challenges and incorporates GI/NBS practices.
2. Identifying systemic issues hindering GI and NBS practices, and providing recommendations for improvements to the high-level policy makers.
3. Sharing experiences and building a network for collaboration among CPI participants.

The Network's diverse work formats included a series of three-day workshops featuring field visits, online meetings, and an additional study trip to Copenhagen for inspirational solutions, and individual counseling (WB 2023).

**Fig. 1:**
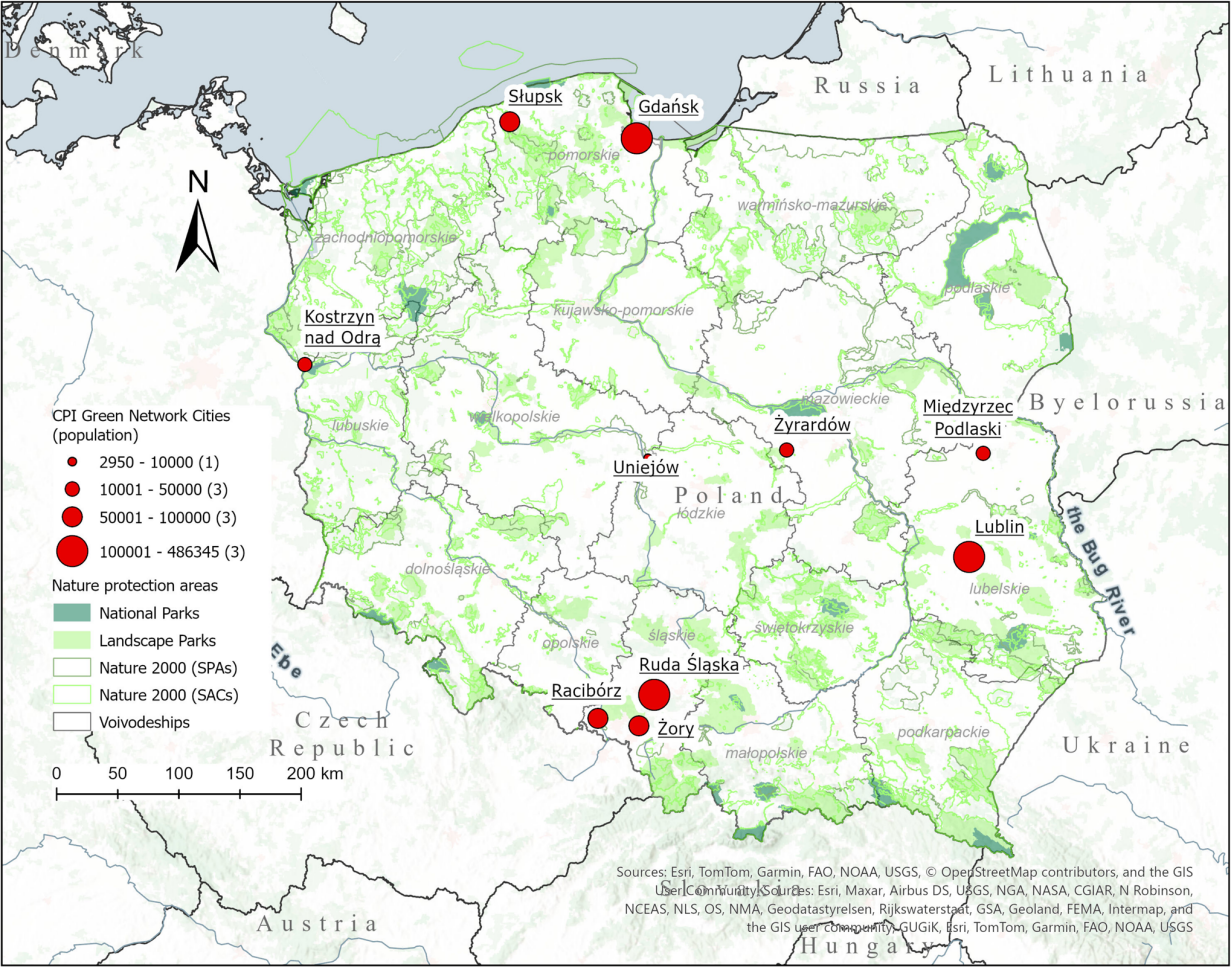
Location of cities participating in the CPI Green Network

Cities were invited to participate in the program based on their applications in an open national call organized by the MDFRP. As a result, the Green Network comprised ten cities from six Polish provinces (voivodeships) (Fig. 1), which varied in area and population size (Table 1).

**Table 1:** Characteristics of cities involved in CPI Green Network. *SMEs - small and medium enterprises. *Source*: Statistics Poland (2022a, 2022b)

City	Area (ha)	Population (2022)	Registered unemployed per 100 working age residents (pers.)	Community social assistance beneficiaries per 10 000 residents (pers.)	NGOs per 10 000 residents (pcs)	SMEs* per 10 000 residents (pcs)
Uniejów	1223	2950	4.5	546	53	1115
Międzyrzec Podlaski	2003	15 557	7.7	616	31	1093
Kostrzyn nad Odrą	4614	17 704	1	298	31	1370
Żyrardów	1435	38 784	5.6	239	37	1492
Racibórz	7501	50 130	2.8	168	33	1063
Żory	6464	61 817	2.1	166	29	1064
Słupsk	4315	86 365	2.7	303	45	1513
Ruda Śląska	7773	131 532	1.3	331	18	865
Lublin	14 747	331 243	4.6	205	64	1567
Gdańsk	26 196	486 345	2	176	50	1876

The Green Network comprised three cities with over 100 000 inhabitants, three with a population of 50 000 to 100 000, and three with a population of 10 000 to 50 000. One city had less than 10 000 residents. Cities participating in the study differ in their socio-economic conditions (Table 1). This diversity is also visible in their urban greenery assets. Selected characteristics of urban green spaces based on available, comparable data are presented in Table 2.

**Table 2:** Selected urban green space characteristics. Highlighted in bold is the highest value for each green space type. *Source*: Statistics Poland (2022a)

City	Recreational parks	Lawns	Street greenery	Green areas of the housing estate	Municipality owned forests
Share of total city area (%)					
Uniejów	**2.79**	0.06	Data not available	0.07	0.08
Międzyrzec Podlaski	0.26	0.06	0.21	0.51	0.10
Kostrzyn nad Odrą	0.24	0.16	0.15	0.59	0.35
Żyrardów	1.88	0.70	0.49	**5**.**87**	0.42
Racibórz	0.48	0.29	0.78	0.65	0.05
Żory	0.04	**0**.**81**	1.38	2.46	0.24
Słupsk	0.99	0.32	1.97	1.36	**4**.**21**
Ruda Śląska	0.73	0.52	1.03	2.70	0.33
Lublin	1.26	0.72	**3**.**59**	3.35	0.01
Gdańsk	0.83	0.62	1.08	2.00	3.99

This diversity of cities makes the study unique because it presents perspectives from not only large cities but also medium-sized and small towns. As a result, the project outcomes may be valuable for many local government units.

## Materials and Methods

**Fig. 2:**
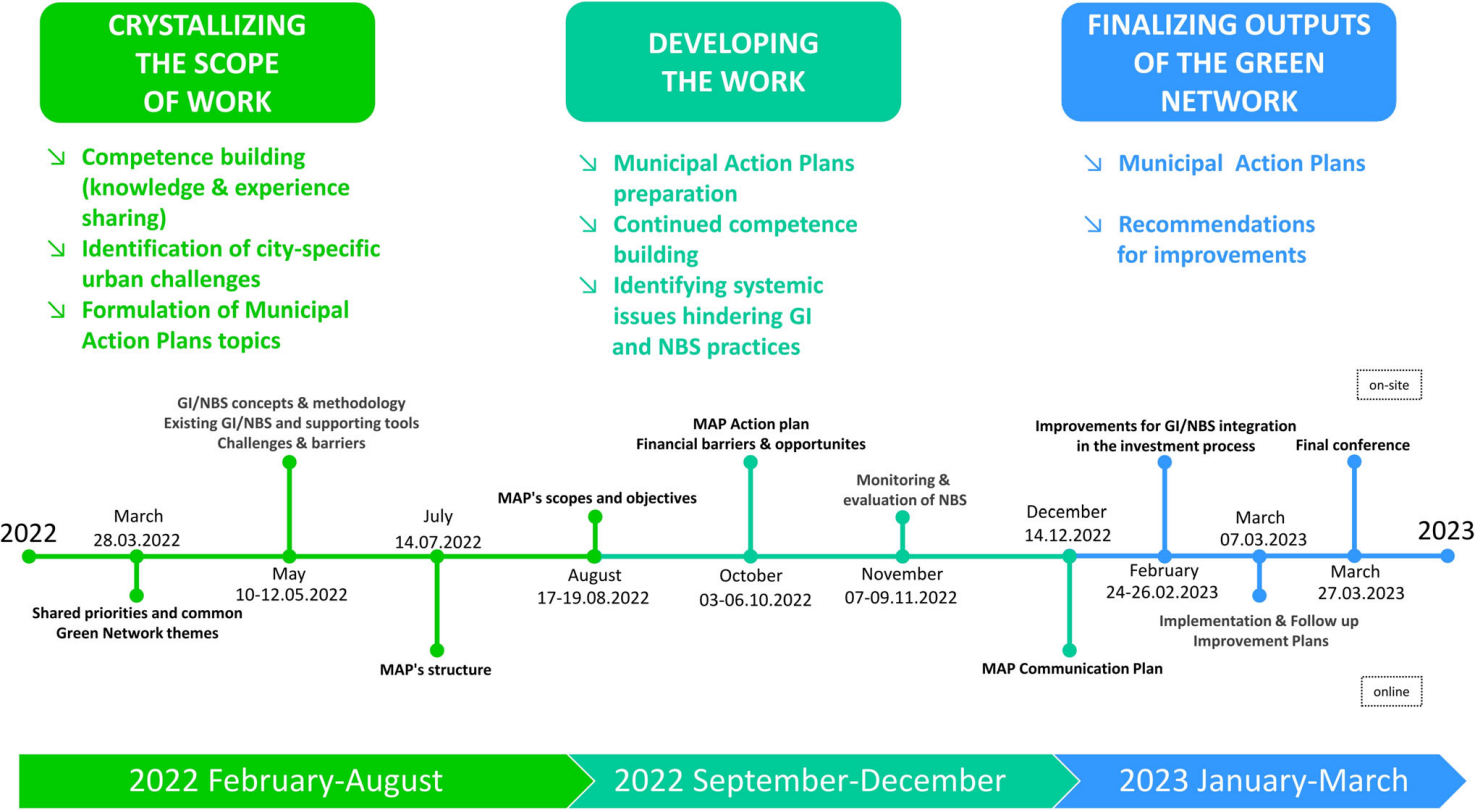
Timeframe and scope of Green Network workshops and meetings

The study objectives were achieved through the analysis of the outputs of the CPI Green Network works developed from March 2022 till March 2023 (Fig. 2). The year-long project implementation allowed for the building of mutual respect and trust among participants, which, according to Allen et al. (2021), is crucial for a successful approach. A detailed description of the scope of work conducted is provided in Appendix S1.

In particular, we made use of the results of the workshops and MAPs prepared by each city. Given the broad scope of the CPI, this paper focuses only on selected outputs of the Green Network that are most relevant to the study’s objectives.

### Workshops as a platform for discussing and exchanging experiences, sharing problems, and finding solutions

The Green Network's initiative consisted of a series of workshops, including five three-day onsite workshops and four online workshops (Fig. 2). Each Green Network city was represented by two delegates, forming a relatively stable group of 20 participants. Cities were represented by practitioners from various municipal units, including those dealing with city strategy and development, climate change, revitalization, maintenance of roads and green areas, environmental protection, greenery and municipal management, and even promotion and fundraising. This highlights the interdisciplinary character of GI/NBS issues.

The workshops aimed to enhance the cities' competencies by sharing knowledge and experience, supporting the identification of specific urban challenges and systemic issues hindering the implementation of GI and NBS, and assisting in the preparation of MAPs (Fig. 2). The workshops were facilitated using scenarios developed by the two authors of this paper, who have backgrounds in environmental planning and management and served as World Bank experts leading the group activities. The onsite workshops were structured to include presentations of scientific concepts by the group leaders, case studies from city representatives on incorporating GI / NBS to address city-specific challenges, and other thematic presentations from representatives of public institutions (e.g., on funding opportunities). Following the presentations, participants engaged in focus group discussions and worked collaboratively using methods tailored to the topics being addressed. The shorter online workshops, in turn, were based on facilitated dialog and open discussion. The detailed scope, sequence of activities, and methods for each workshop are presented in Appendix S1.

Through the workshop series, the participants were introduced to the concepts of GI and NBS along with their potential benefits. Identification of key urban challenges and barriers to a systemic approach to GI and NBS was conducted based on participants' individual experience from their daily work. Challenges were identified within municipalities and grouped according to the 10 pre-defined urban challenges (Raymond et al. 2017, modified). Barriers were assigned to one of four pre-prepared categories: organizational, legal, substantive, and financial. Visually arranging problems (flipcharts, sticky notes) created a starting point for further discussions and supports the process of diagnosing systemic constraints and joint search for solutions.

City’s needs and recommendations for improvements were gathered throughout the project, drawing on individual participants' inputs as well as focus group discussions, during which various opportunities for enhancement were identified. The materials gathered during group work contributed to a deeper understanding of the topic by incorporating diverse perspectives from practitioners with various backgrounds and experiences in city management. Such an approach enabled the co-production of knowledge, defined as iterative and collaborative processes involving diverse types of expertise, knowledge, and actors to produce context-specific knowledge and pathways toward a sustainable future (Norström et al. 2020). According to the four main process principles formulated by Norström et al. (2020), the process was context-based, pluralistic, goal-oriented, and interactive. Such an interactive formula of the workshops is also applied in other studies involving stakeholders (Tusznio et al. 2020; Sagie and Orenstein 2022).

### Municipal Action Plans as ‘roadmaps’ for addressing city-specific challenges

MAPs constituted outputs of the CPI Green Network in the form of roadmaps presenting each city’s approach to tackling its sustainable urban challenges with the application of GI and/or NBS (Table 3).

**Table 3:** Topics of Municipal Action Plans (MAPs) developed by Green Network cities with proposed GI/NBS-related actions. Type 1 Spatial actions in the urban space: 1.1. Protection of existing urban green of high nature value; 1.2. Increasing the quality or multifunctionality of urban green spaces; 1.3. Creating new urban green areas/objects; Type 2: Actions supporting systemic urban green uptake and management; 2.1. Enhancing multi-stakeholder collaboration; 2.2. Increasing knowledge and awareness; 2.3. Developing guidance or tools

City	MAP territorial scope	MAP topic	Proposed GI/NBS-related action type
Gdańsk	City	Popularization of surface unsealing and re-wilding urban spaces	1.2. Pilot design of a schoolyard space 2.2. Popularization of surface unsealing and re-wilding urban spaces (brochure)
Kostrzyn nad Odrą	City center districts	Development and revitalization of small-scale green areas, developing cooperation with property managers and public education	1.2. Revitalizing existing green spaces 1.3. Planting urban trees 2.1. Framing collaboration with property owners and managers 2.2. Awareness-raising education and popularization campaigns
Lublin	City center districts	Introduction of new green elements, improvement of existing greenery, promotion of NBSs, education and involvement of residents in local projects	1.2. Developing a concept for green space restoration, revitalizing and converting existing squares into small green spaces, renewal of containerized plantings, widening existing tree pits, implementation of rain gardens, protecting green areas along road corridors from vehicle damage and trampling 1.3. Desealing surface, planting urban trees, climbing plants, greening transportation stops 2.1. Engaging residents in local initiatives 2.2. Education and promotion of NBS 2.3. Promoting the "Urban Green—Standards" guidelines among private and public investors
Miedzyrzec Podlaski	City	Creating new small-scale green solutions; improve quality of existing green spaces; educational and resident involvement actions	1.2. Revitalization of degraded areas into green spaces 1.3. Increasing the area of green spaces in highly urbanized zones (e.g., planting trees and bushes, desealing surface, introducing parklets, small gardens, greening transportation stops) 2.1. Engaging residents in local initiatives 2.2. Educational activities
Racibórz	City	Introducing NBS in the backyards along with resident involvement activities and wide education and training	1.2. or 1.3. Creating a conceptual project for a pilot courtyard adapted to climate change and attractive to residents 2.1. Establishment of the interdepartmental team supporting sustainable development 2.2. Capacity building of municipal staff (training) 2.2. Enhancing the courtyard space with an educational boards, thematic workshops for residents during public consultations
Ruda Śląska	City	Creating a green corridor linking the city center areas and the Bytomka valley with green pedestrian and cycle routes (connecting the northern districts with neighboring cities)	1.1. Protecting the most valuable natural elements of the city and maintaining the continuity of ecological system of stream valleys and water reservoirs 1.2. Shaping existing managed green spaces, especially in areas with a high degree of environmental degradation 1.3. Creating a network of green wedges connecting different city districts, linking river valleys, and establishing pedestrian and cycling routes
Słupsk	Central and western city districts	Blue-green infrastructure and small retention facilities for stormwater management	1.2. or 1.3. Concept for the development of green areas and small retention facilities 1.2. Utilizing existing green spaces to implement blue-green infrastructure (BGI) projects and adapting them for water retention, modernization of existing green spaces 1.3. Desealing of surface and introduction of new green spaces 2.1. Establishment of a team for developing standards for the design and maintenance of public spaces 2.1. Engaging residents in local initiatives 2.2. Supporting pro-environmental attitudes among children and promoting NBS 2.3. Introducing guidelines for investors on rainwater retention and biologically active surface areas
Uniejów	Part of the city (urban and spa zone), in the historic part of the town square, in the outlying streets, buildings, domestic spaces	Enriching the urban area with green belts and leisure zones and encouraging residents to introduce greenery on their properties along the streets and in their home gardens	1.2. Revitalization of degraded large urban areas into new green spaces 1.3. Increasing urban green including: planting trees and shrubs along roads, desealing surface and planting new vegetation, creating parklets, greening transportation stops, creating small urban gardens 2.1. Engaging residents in local initiatives
Żory	Post-industrial area	A comprehensive reclamation and adaptive reuse of former mining infrastructure, assigning new socio-economic and educational functions, with the use of aquaponic systems and the introduction of green spaces	1.3. Development of an architectural concept that integrates environmental and socio-economic measures such as production of organic food using a closed water circulation system, planting native tree and shrub species, introducing small-scale water retention, and implementing extensive green roofs, creating conditions for plant pollination, piloting and testing innovative solutions in urban agriculture and experimental education 1.3. Planning new green spaces as community integration and recreation areas 2.1. Accelerating startups in innovative urban agriculture, preparing a skilled workforce for business transformation in the region, supporting the creation of social economy entities 2.2. Raising public awareness about climate change and its impact, increasing knowledge about organic farming, urban agriculture, zero waste, and slow food concepts
Żyrardów	Central revitalized district	Systemic greenery guidelines for urban courtyards regeneration with educational program for residents and preparation for financial mechanisms	2.1. Supporting civic initiatives aimed at improving environmental quality 2.2. Strengthening climate and environmental awareness and responsibility among residents 2.3. Development of general design guidelines for the planning of neighborhood spaces

Each MAP followed a template that structured the process of preparing a city’s solution(s). All MAPs include the following components: a description of identified challenge(s), goals, and solutions planned to achieve these goals; a description of activities to be implemented; stakeholders mapping and an outline of a communication strategy; and an assessment of resources (human, technical, and financial) required to implement actions and achieve MAP’s objectives as well as monitoring plan and project risk assessment.

**Fig. 3:**
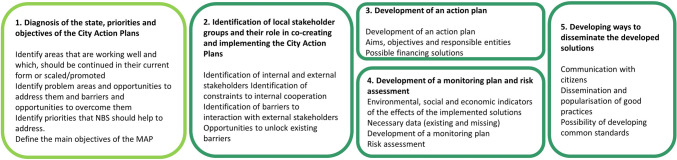
Framework for MAPs preparation

MAPs were prepared individually by each of the two-person teams representing the cities participating in the project. WB experts provided support and guidance to maximize practical and educational benefits for the cities involved according to the MAP preparation framework (Fig. 3). In some cases, the role of the experts was to support cities in embedding GI or NBS (when necessary) and to highlight multiple potential benefits of the proposed projects—some of which had not been initially recognized by the participants. In other cases, cities were advanced in project development incorporating GI or NBS and did not require substantial guidance.

Despite their structural commonalities, individual MAPs differed (for example, in the thematic and territorial scope—such as a city, district, or smaller specified areas—and the level of details of the description of a particular challenge (e.g., documented by data) and solution (e.g., general concept or technical detailed project). These differences arose from the unique challenges faced by each city and the level of advancement in developing its own solutions. The synthetic presentation of the proposed GI/NBS-related actions reflects the dual nature of interventions: spatial actions in the urban space aimed at protecting, improving, or creating GI/NBS and actions supporting systemic management aimed at developing collaboration, knowledge, and other tools such as design guidelines or standards (Table 3).

## Results

### Key urban challenges and potential of GI and NBS to address them

The practitioners' perspective on challenges is typically viewed through the lens of the need for improvement in urban space, with GI and NBS commonly regarded as key tools for achieving such enhancements.

In the initial stage of the project, Green Network participants identified the challenges their cities were facing, which were later structured within a framework of ten challenges developed by Raymond et al. (2017), adapted to the Polish context.

Among the challenges, city representatives most frequently indicated those related to quality of the urban space, green space (infrastructure) management, and participatory planning and management (Table 4).

**Table 4:** Challenges indicated by the city representatives. *Cities were free to indicate as many challenges as necessary; the individual challenges were grouped into presented themes; as a result, the number of mentions (84) is higher than the number of cities (10)

Challenges themes	Prevalence of mentions*
Quality of the urban space	21
Green infrastructure management	15
Participatory planning and management	9
Public health and well-being	8
Air quality	8
Effects of climate change	6
Water management	6
Economic opportunities and green jobs	5
Other	4
Social cohesion and justice	2

The urban space quality challenges reflected various issues from socially oriented, such as the lack of a center for social life or lack of attractive public spaces, through more environmentally oriented such as the presence of degraded and neglected spaces that once presented high quality and the occurrence of brownfields or mining damages. Simultaneously, practitioners saw high potential in GI and NBS that can contribute to positive changes by creating public and green spaces for residents that will address their needs, renovating or renewal of existing greenery, green development of post-mining areas and changing the image of those areas. As challenges to employing the above potential, practitioners identified appropriate compensation for greenery removed due to investment (discretionary), the development of common standards for plantings, as well as ensuring effective regulations and financial resources for green space protection. The urban space quality challenges were therefore closely associated with challenges in overcoming barriers contributing to the current unsatisfactory state.

Within green space management, issues that emerge relate to shortages in spatial structure (too few green spaces in the city center, lack of a coherent concept of green city development, lack of connection between urban spaces and disorder in space, the collision of the economic, protective and social functions of forests or mortality of animals). Again, an important part of the indications concerns management challenges (e.g., agreeing on standards for establishing and maintaining green areas, ensuring financial resources for their maintenance, and the adequate integration of new investments with existing or planned greenery).

The challenges related to participatory planning and governance included low involvement of residents in public consultations and spatial planning, difficulties in engaging residents in fair transformation, a lack of conviction of officials and councilors about the value of consultations, and low NGOs involvement in cooperating with the city. Additionally, practitioners indicated challenges related to the process of running and participating in the collaboration with different actors.

Air quality is a concern for many cities. The challenges in this area include not only low air quality, often resulting from low emissions, but also the lack of planned green spaces and trees, the absence of a coherent and attractive network of bicycle paths to promote zero-emission transport, and the lack of rational spatial planning. Air quality issues are also connected to securing financial resources for implementing the Low-Emission Economy Plan.^2^

The health and well-being challenges were linked with urban spaces that are not friendly for residents, the presence of neglected yards, the absence of attractive public spaces, and low air quality. The indicated potential for improvement included local solutions such as developing and renovating housing estate greenery, urban farming, city orchards, and producing healthy food.

Effects of climate change, such as strong winds, heavy rains, and high temperatures, received specific attention from certain municipalities due to their consequences, including flash floods, urban heat island, reduced lifespan of trees, and tree withering.

Water management turned out to be just as important as addressing the effects of climate change. Representatives from cities emphasized a range of issues, including excessive sealing of surfaces, anthropogenic transformations of the coastal belt (coastal protection), degraded river valleys, fortifications of channels, insufficient retention, and insufficient action to counteract the effects of drought.

Slightly less attention was given to economic opportunities and creating green jobs. In this respect, practitioners noticed that not all cities have own specialized municipal unit dealing with greenery maintenance, the challenge of generating competitive green jobs in maintaining green spaces, the lack of green tourist products, and the failure to promote the creation of green investment areas. They also highlighted the need for economic stimulation of the area in synergy with their greening activities.

The lowest interest was expressed in social justice and cohesion challenges, with only one indication of tackling social exclusion and facilitating professional reintegration.

**Fig. 4:**
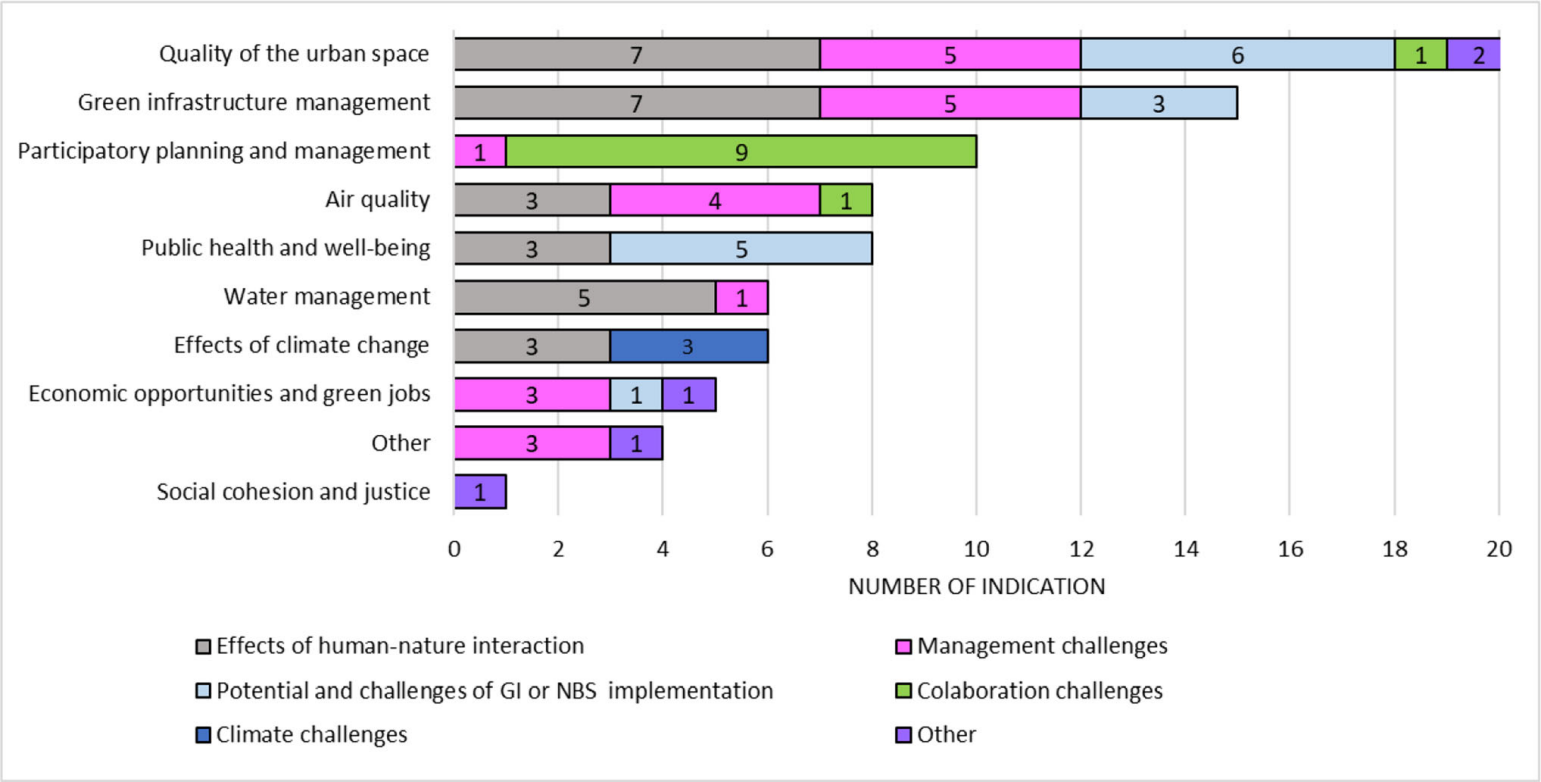
Sub-types of ten urban challenges. Cities were free to indicate as many challenges as necessary; therefore, the number of indications (84) differed from the number of cities (10)

The most frequently identified challenges can be attributed to the effects of human-nature interactions, including human pressure (28 indications). Highly visible is linking the quality or state of the urban space with management challenges (22). In addition, a simultaneous focus on the potential of GI/NBS and the challenges of their implementation should be indicated (15). Next are challenges related to collaborations with different actors in a participatory manner (11) (Fig. 4).

The challenges identified by cities are often interconnected, and a broader view reveals their environmental and human context. Results showed that practitioners strongly link spatial and environmental urban challenges with legal, institutional, and process-based challenges. Consequently, participants of the Green Network perceived the implementation of GI and NBS as complex and demanding.

### Barriers to the systemic approach to implementation of GI and NBS

Among the barriers hindering the greening of urban spaces, city representatives identified the following during focus group discussions: legal aspects that hinder and limit municipal actions; organizational issues (including cooperation within and beyond city administrations); substantive limitations (in terms of knowledge and skills); and financial restrictions.

#### Legal barriers

Among legal barriers, participants indicated those that hinder action on the ground. These included ownership laws, which make it difficult to operate outside city-owned areas, the need to cooperate with infrastructure operators, and the unregulated legal status of real estate, e.g., post-mining property. Other obstacles included insufficient regulations for protecting green spaces and trees, such as inadequate fines for radical pruning and a lack of regulations enabling drivers to be penalized for parking in green areas when they are already degraded. Additionally, there are no general guidelines for nature compensation procedures, and there is unspecified liability for damage caused to the environment.

#### Organizational barriers

City representatives identified various organizational barriers, including incoherent distribution of responsibilities, problems with information flow, low personal involvement of employees in city affairs, and insufficient vertical and horizontal cooperation. Staff shortages were also mentioned, indicating a need for more qualified experts and appropriate units. There are noticeable differences between large and small cities in terms of organizational barriers. Larger cities face the 'silo effect' among city units, while smaller ones operate with limited human resources.

When it comes to outside-organizational barriers, difficulties result from insufficient awareness among residents and councilors and social attitudes expressed in resistance to change or cooperation. Commonly mentioned obstacles include a lack of partners for cooperation and the challenge of creating a network for collaboration. This includes insufficient activity from local partners such as NGOs or leaders of local societies, as well as lengthy administrative procedures with other authorities involved in the administrative process.

#### Substantive and financial barriers

The difficulties in a systemic approach to planning, design, execution, and maintenance of GI and NBS are compounded by the fact that some tasks require an innovative approach beyond the standard competencies and responsibilities of council employees. In particular, complexities arise in reconciling the interests of different stakeholder groups and managing conflicts between building, transport infrastructure, and green space planning and design. Additionally, small towns have limited access to specialized experts and tools such as those in rainwater management, unlike larger cities that have established special units for such purposes. For example, the municipal company Gdańskie Wody Sp. z o.o. in Gdańsk handles rainwater management, which smaller towns lack.

Moreover, financial resources heavily impact the availability of specialist staff and experts (who tend to have high rates that are not easily affordable for municipal administration). The high cost of expert services can be unaffordable, particularly for smaller towns.

Another issue is that GI and NBS are not considered municipal priorities when it comes to spending, which makes their budgets susceptible to cuts. New green spaces are often viewed as requiring ongoing maintenance costs rather than generating income. This situation is exacerbated by inflation and the lack of budget increases, resulting in funding that falls short of meeting actual needs.

### Recommendations for integrating GI and NBS in urban investments

The group work, shaped by the diverse contexts and conditions of the participating cities, resulted in a broad spectrum of proposals for better integrating GI and NBS into urban initiatives. Since the aim of the CPI was to support both cities and central administration in the relevant thematic areas, the recommendations were clustered into two levels of governance: local and national. At both levels, some recommendations were commonly shared across cities, while others were specific to individual contexts. At the local level, the recommendations were presented distinctly for the planning and design phases of GI and NBS, as well as for their execution and maintenance phases. This structure reflects the specific needs identified by participants in relation to the challenges and barriers inherent in each phase of project cycle.

#### Planning and design phases: Local-level recommendations

At the planning and design phase—once the investment location is determined—municipal practitioners recommended several key actions. These include conducting an inventory of environmental conditions, such as existing greenery, wetlands, microclimate, soils, slopes, and water regime, to serve as a baseline for further action. They also suggested formalizing the inventory requirements through appropriate legal regulations. In addition, participants advised carrying out a greenery audit to support designers. This would involve an assessment of existing greenery by a greenery supervision inspector. A standard, which determines that the potential of greenery is well used, would serve as a valuable references for such assessments. Practitioners also proposed developing specific guidelines for greenery within the investment process or adopting “open” standards already implemented in cities like Wrocław and Kraków. These standards would address aspects such as the size and quality of new plant material and protection measures during construction works.

Further recommendations included the systemic integration of greenery across key municipal documents such as the Municipal Revitalization Plan, Climate Adaptation Plan, Environmental Protection Program, and local development or transformation plans. City representatives emphasized the need to strengthen the role of greenery in the decision on environmental conditions of approval to a project, architectural concepts, and setting accessibility standards. They also suggested adopting a procedures manual—attached to the tender specifications. This manual could include, for example, a list of recommended plant species suitable for the area, taking into account maintenance costs. Participants emphasized the importance of incorporating financial assumptions for the maintenance of newly planned green spaces. Also, opportunity is seen in innovative solutions for funding, such as citizens' budget, within which local communities propose projects and vote for the implementation of the most wanted.

Additionally, practitioners recommended establishing an interdepartmental team, comprising members from the investment department and the greenery conservator, which would meet regularly to coordinate efforts. Moreover, they called for improved data flow, through data digitization, regarding the plans of individual departments, potentially stored in the cloud as part of a broader digital municipality initiative.

#### Execution and maintenance phases: Local-level recommendations

City representatives outlined several recommendations related to the execution and maintenance phases. First, they emphasized the importance of ensuring independent supervision over the work of the greenery inspector, particularly in terms of adherence to greenery protection conditions during the investment process and compliance with the project. In this context, they strongly advised against combining the role of designer with the function of supervisory inspector, due to the potential conflict of interest. Additionally, they suggested considering a strategic decision on whether to entrust the maintenance of green areas to an internal municipal unit or to outsource it to an external entity, highlighting the trade-off between maintenance costs and control over the quality of services. Finally, they recommended strengthening the engagement of the local community. This could be achieved, for example, by organizing grant competitions for local groups to support activities such as caring for newly introduced vegetation and monitoring greenery maintenance. Additional opportunities include collaborating with local businesses and creating low-maintenance green spaces, allowing savings to be reinvested in new green initiatives.

#### Recommendations at the national level

At the national level of governance, practitioners have developed a set of recommendations, acknowledging the importance of broader-scale frames that guide local interventions. First, they suggested incorporating greenery protection conditions into legal regulations—specifically, by introducing provisions that define requirements for the development of green areas, similar to development conditions currently established in administrative decisions for buildings.

Second, city representatives also emphasized the need to hold designers accountable for proposing greenery appropriate to specific environmental conditions. For instance, designers face consequences when a building collapses, but they bear no responsibility if poorly planned greenery withers.

Third, practitioners recommended disseminating catalogs of good practices, including the introduction of guiding documents at the national level for urban areas. Another important suggestion was the adoption of nationwide standards to support the development of a common protocol for the protection of greenery components. These standards would address issues such as the protection of greenery during the investment process (e.g., recommended nationwide standards for tree-cutting methods), greenery maintenance practices, and the simplification of administrative procedures.

## Discussion

### Global challenges from cities’ perspectives

Only a subset of urban challenges can be addressed through GI/NBS, and their significance is influenced by contextual factors such as urban size and geographical location (Babí Almenar et al. 2021). The key challenges identified by Green Network participants mainly relate to the consequences of human pressure or human–nature interactions, which GI and NBS are well positioned to tackle. Taken together, these challenges underscore the persistent problem of spatial competition between urban greenery and investment.

The representatives of Polish cities indicated the rich list of challenges that can be addressed by GI or NBS, highlighting their multifunctionality (Babí Almenar et al. 2021). Most frequently mentioned were urban space quality and green space management challenges, as well as participatory planning and governance, which is not surprising, given the thematic streams defined within CPI and the profiles of practitioners who represented mainly development and environmental units. A characteristic finding is that practitioners viewed urban challenges in close connection with the challenges of overcoming the barriers underlying the current unsatisfactory state.

The challenges of social cohesion and social justice were identified sporadically, under a specific context (e.g., transformation of post-mining areas). Although these issues are not typically recognized as primary objectives of urban greening initiatives, the outcomes of such efforts are intended to fulfill the needs of residents and contribute positively to both social cohesion and justice through various health benefits (Jennings and Bamkole 2019; Cardinali et al. 2024). This misalignment may stem from practitioners’ understandings of the issues, which can diverge from academic classifications, from the practice of placing social needs at the core of urban action while still viewing them as separate from social problems, and from the ambiguous and often inconsistent understanding of social cohesion (Chan et al. 2006; Schiefer and van der Noll 2017) and social justice (Lager et al. 2023; Biesbroeck et al. 2024). A more deliberate inclusion of these issues in urban greening emerges as another area for consideration within a systemic approach, particularly given their close links to participatory planning and collaborative management (Mauer et al. 2023) and to public health and well-being (Jennings et al. 2024)—both well-recognized urban challenges in Poland.

To strengthen cities in addressing various urban challenges through GI and NBS, the European Union advocates for their experimentation and implementation. It fosters cooperation and the exchange of experiences between cities and disseminating good practices (Faivre et al. 2017; Davies et al. 2021), which is particularly beneficial within similar contextual conditions and challenges (Babí Almenar et al. 2021). Yet, despite continued support, there is still significant potential for expanding the use GI and NBS, even in developed countries (Zwierzchowska et al. 2022).

European Union support efforts toward a green urban transition by increasingly recognizing GI and NBS in its policies, financing numerous research and innovation projects, and developing various initiatives and studies that promote this approach (Xie and Bulkeley 2020; Balzan et al. 2021). Particular role plays policies and measures developed to achieve the European Green Deal (EC 2019). For example, cities take center stage in the EU Biodiversity Strategy for 2030, being one of the European Green Deal pillars (Zulian et al. 2022).

Both the main thematic streams of the Green Network initially identified by the MDFRP and the specific individual urban challenges selected by cities indicate that the call for implementing GI and NBS, as embedded in international initiatives (e.g., SDG goals) and EU policies, addresses the vital challenges of Polish cities despite their diverse social, environmental, and economic contexts. City representatives participating in the Green Network highlight that further incentives and support from supra-municipal levels are necessary. This is especially important for legal, organizational, knowledge-related, and financial conditions that require developing regional, national, or European solutions.

### Key barriers in municipal practice

As demonstrated, Polish cities encounter various obstacles in the systemic development of GI and NBS, which together create a complex environment that hinders their uptake and mainstream. The barriers include inadequate legal frameworks, shortages in organizational collaboration (both within and outside the city administration), limitations in knowledge and skills (of actors involved in the greening process), and financial constraints that affect institutional capacity. While legal barriers (e.g., ownership law) and certain outside-organizational barriers (e.g., insufficient awareness among residents and councilors, limited engagement of local partners, lengthy administrative procedures with other authorities) are common across all cities, inside-organizational barriers vary by city size. Smaller cities, in particular, report limited human resources, whereas larger cities experience the “silo effect.” Smaller cities also placed stronger emphasis on substantive barriers resulting from limited access to experts, and although all cities reported financial barriers, these were especially pronounced in smaller ones.

Similar limitations have also been noticed in other countries. In France, professionals working on NBS indicate that deficits in knowledge, political will, financial resources, and regulations are the main barriers (Duffaut et al. 2022). Limited collaborative governance, knowledge, data and awareness challenges, low private sector engagement, competition over urban space, insufficient policy development, implementation and enforcement oriented at NBS, insufficient public resources, and citizen engagement challenges stem also from cities’ experience from Germany, Hungary, the Netherlands, Spain, Sweden, and the UK (Dorst et al. 2022).

Literature reviews indicate an even broader range of barriers (e.g., Sarabi et al. 2019; Perrault 2022; Martin 2025). Sarabi et al. (2019) identified inadequate financial resources, path dependency, institutional fragmentation, inadequate regulations, and uncertainty regarding the implementation process and effectiveness of the solutions. Lack of professional expertise and resources, lack of public knowledge, performance uncertainty, contextual uncertainty, fear of negative consequences, fear of operational unknowns, insufficient interactions between key players, structural silos, path dependency, temporal mismatches, spatial constraints, valuation, and financial constraints—was reported by Perrault (2022). According to Martin et al. (2025) among the most prominent governance barriers are those related to social factors (lack of expertise and knowledge, equity issues, stakeholder engagement and conflicts, risk aversion) followed by legal (path dependency, lack of supportive policies, sectoral and administrative silos, and land ownership and availability), economic (the lack of evidence on NBS delivery, performance and co-benefits, lack of funding and high costs of NBS, maintenance costs), political factors (the lack of political will and long-term commitment), and environmental/ecological factors (the potential negative impacts or ‘disservices’ of NBS).

Against this background, the barriers to adopting a systemic approach to GI or NBS identified by Polish practitioners are broadly consistent with findings from other studies. Interestingly, some issues not initially recognized as barriers emerged in the recommendations as important for strengthening a systemic approach, such as the need for closer cooperation with the business sector. Additionally, although some barriers—such as uncertainty or fear—were not explicitly reported, they were mirrored in practitioners’ reluctance toward evaluation and monitoring.

Despite the growing body of literature on barriers to NBS implementation and mainstreaming, practitioner-based studies remain limited, and the comparability of results is constrained by the diversity of analytical frameworks and classification schemes used across studies and contexts. This underscores the need for further work toward establishing a common approach.

### Pathways for systemic integration of GI or NBS

In addition to identifying barriers in the systemic approach to GI and NBS development, municipal practitioners also see several potential opportunities for improvement. One of these is the need for stronger interdisciplinary planning and design of green spaces, as evidenced by shortcomings in the coordination of green space projects in Polish municipalities—an issue highlighted both by the Green Network participants and by the findings of Legutko-Kobus et al. (2024). Duffaut et al. (2022) also emphasize the proposed solutions of transdisciplinary research disciplines, as well as on-field collaboration between all actors involved in NBS in cities. However, city-level governments require paradigm shifts for understanding how institutional change can be achieved (Adams et al. 2024) toward more systemic GI/NBS inclusion. The experience of Green Network reveals that within city administration, key partners in fostering the greening process are representatives of investment departments responsible for planning, implementing, and managing new investments. Additionally, to break away from the business-as-usual approach and seek new and innovative paths to GI/NBS in municipal actions, staff knowledge, skills, and commitment are crucial resources. However, such a complex approach for greening the cities requires “making the case, building coalitions, breaking down silos, solving thorny problems, championing innovative and integrated solutions, and demonstrating performance” (Jonhston et al. 2013). These tasks require green city leader skills to act as an agent of change within the municipality office and in the community (Jonhston et al. 2013) as well as NBS-specific expertise (Martin et al. 2025).

To enhance the uptake and mainstream of GI and NBS in urban areas, stronger support from political decision-makers and their reflection in urban policy is vital. This finding is not surprising, as integrating NBS into urban policy and planning is new but progressing across European cities (Kauark-Fontes et al. 2023). Nevertheless, the process remains challenging because efforts to address urban challenges might compete with one another, and differing approaches to sustainable urban development—such as the compact city model—can further constrain urban greening efforts (Haaland and Konijnendijk van den Bosch 2015).

Collaboration with different stakeholders is seen as a management strategy for improving performance in addressing complex issues (O’Leary et al. 2012) and shared understanding of problems (Wickenberg et al. 2021) that cannot be solved or easily addressed by a single organization (Agranoff and McGuire 2003). The inclusive stakeholder engagement and true co-design can facilitate this process (Martin et al. 2025). However, city representatives have emphasized the difficulties associated with this collaborative approach.

The concept of the silo mentality emerged as another key issue during Green Network meetings. De Waal et al. (2019) and Kauark-Fontes et al. (2023) emphasized that it has long been recognized as a problem that needs to be addressed. However, transitioning from the silo approach to collaboration requires acquiring new skills to engage partners and bring multiple stakeholders together. Individuals and their collaborative skills play an essential role in this process (O’Leary et al. 2012), especially in Poland, where the operational possibilities of participation are still developing (Kotus 2013). This is also crucial in light of identified shortages in awareness and social attitudes for cooperation, resulting in a lack of partners for collaboration. Barriers related to limited social engagement and civic passivity were also reported by representatives of smaller towns (Legutko-Kobus et al. 2024), highlighting the need to reshape relations between citizens and governing institutions that might suffer from mistrust (Mercado et al. 2024). As Hölscher et al. (2024) propose, strengthening collaborative governance through co-production calls for adopting a tailor-made approach for inclusive, place-based engagement, embedding open-ended co-production with long-term benefits, and fostering new roles and relationships to sustain co-production practices. To facilitate this process, Frantzeskaki et al. (2020) suggest that cities can invest in tailored and targeted capacity building programs, and institutional spaces must allow for collaborative learning and partnerships. Additionally, showcasing examples of successful partnerships from other cities can boost promotion and investment in NBS (van Ham and Klimek 2017).

For urban greening purposes, cooperation with contractors is also crucial. Finding contractors with experience in delivering NBS projects is difficult (Mačiulytė and Durieux 2020), and the outcomes of contracting out green space management related to service quality can be unsatisfactory (Lindholst et al. 2017). The recommendations proposed by city practitioners addressed the need to strengthen contractors' responsibilities and robust control of their work. This goal can be achieved by utilizing various tools, including service specifications, pricing and payment instruments, monitoring, safeguards, adjustments, and organizational tools (Lindholst 2009). In reference to the above, creating generally applicable standards in green space planning, design, and management is essential. A standard is a published document that defines an agreed-upon, repeatable method for performing a task. It includes technical or specific criteria for consistent application as a rule, guideline, or definition (BSI 2012). Some cities have independently developed standards for planting trees and maintaining greenery, such as Kraków, Wrocław, Warszawa (Jedliński 2018), Lublin (2022) and Poznań (2022). Setting standards for green space accessibility is also noted as an important tool and procedure (Rubaszek et al. 2023). These approaches have recently emerged in Polish municipal strategic and planning documents (e.g., Gdańska Polityka Zieleni (Gdańsk Green Policy), 2023; Poznań SUiKZP (Study of Condition and Spatial Development Direction of Poznań), 2023) (Skibińska 2025). In addition, the recent Act on Spatial Planning and Development changes allow voluntary standards for public green space availability (Act 2003). The ongoing changes manifest that cities in Poland are embracing the challenge of setting standards and voluntarily implementing them, recognizing their significance in greening cities. However, these are just a few municipalities; most do not have such solutions.

Financial resources are essential for the realization and maintenance of GI and NBS. These actions are primarily funded through limited municipal budgets, which have been indicated as a persistent barrier for their widespread. This is particularly evident in smaller towns (Legutko-Kobus et al. 2024). To leverage other financial resources for NBS implementation and overcoming the budget shortfalls, Biasin et al. (2024) suggest strategies such as fostering innovative and alternative financing models and collaboration, including co-financing, with engagement of corporations, the insurance sector, real estate firms and water utilities. The review of financing approaches that can be used to deliver GI or NBS in urban areas is also complied in Trinomics and IUCN (2019). Toxopeus and Polzin (2021) also stressed the overarching barriers of NBS finance, pointing to the shortages in coordination between private and public financiers and in integrating NBS benefits into valuation and accounting methods. In this regard an evidence based on NBS performance and their co-benefits, including quantitative cost–benefit analyses, can be helpful (Martin et al. 2025).

A part of the systemic development of GI and NBS is monitoring and evaluating undertaken activities and their impact on targeted urban challenges. However, practitioners have yet to appreciate the importance of monitoring the impacts of urban interventions. While it is crucial to assess the success of these projects (Bannan et al. 2022) and provide robust arguments showing the multiple benefits of GI and NBS to inform the political leaders and local communities—the value of such feedback was underrated by the city representatives. The monitoring assessments were seen as a bugbear in case of failure to achieve the intended goal rather than as a tool for improving future activities and reducing uncertainties (Perrault 2022).

Finally, the cities' representatives also formulated their recommendations for improving specific regulations. The detailed proposals for changes were gathered during the Green Network work and formulated into an Improvement Plan (WB 2023).

## Conclusions

GI and NBS are increasingly recognized as tools to address numerous urban challenges. Despite the benefits acknowledged by municipal practitioners, the experience of Green Network showed that the systemic approach to greening the city still needs to overcome a set of barriers. These barriers include weak legal frameworks, insufficient organization and collaboration, limited knowledge and skills among stakeholders, and financial constraints hindering institutional capacity. Simultaneously, city office practitioners proved to be full of ideas for potential improvements that, from their perspective, would facilitate systemic greening in the cities.

The results revealed that to enhance systemic transformation at the municipal level, there is a need to strengthen the position of GI and NBS in urban policies and investment processes while improving coordination across municipal departments to overcome a silo approach. Particular attention is required for smaller towns, which often lack the expert knowledge and specialized solutions needed to tackle modern challenges like climate change. Also, continuous capacity building through collaboration with citizens and NGOs, fostering skills for co-creation and co-management, is highly needed. Next, developing standards and tools is essential to better manage and protect green spaces, alongside finding cost-effective solutions such as low-maintenance designs and citizen involvement. Finally, innovative financial approaches, including private sector engagement, are necessary to support the integrated uptake of GI and NBS beyond demonstration or single projects toward widespread systematic adoption.

To enhance the development and engagement of both political leaders and local communities in GI and NBS, it is essential to monitor and evaluate the initiatives undertaken. This evaluation, though sometimes underrated by practitioners, provides crucial arguments that demonstrate the multi-beneficial nature of GI and NBS.

Practitioners often point to the need for specific tools and solutions to support their actions' effectiveness in greening cities. However, it must be remembered that the complex and dynamically changing reality often escapes rigid patterns and requires an individualized approach. Therefore, developing tools and solutions that are adaptable locally and meet cities' needs is undoubtedly an open field for further work. One of the approaches to foster this process and the transfer of good practice is knowledge exchange among the cities.

The findings from the Green City Network experience align with the growing trend of mutual learning through networks or partnerships. These networks enable cities to share experiences and support each other in addressing similar challenges. Therefore, we add to the discussion on the significance of city-to-city networks, which, according to Meagher et al. (2021), can enhance individual city responses to challenges and foster better collaborations at the state, city, and local levels.

## Supplementary Information

Below is the link to the electronic supplementary material.

Supplementary file1 (PDF 937 KB)

## Footnotes

1 www.pim.gov.pl.

2 The Low-Emission Economy Plan is a strategic document that sets the direction of development toward low emissions. It is prepared by municipalities that want to apply for funding for projects related to energy efficiency and renewable energy sources. It is the basis for applying for external funds to implement the tasks formulated in the plan.
