# Supplementary material for: Systemic approach to green infrastructure and nature-based solutions uptake: Insights from the Polish cities
Source: Ambio. 2026 Mar 24;55(8):1898–917. doi: 10.1007/s13280-025-02336-0 (PMC13319540; doi:10.1007/s13280-025-02336-0)
Supplement: Supplementary file 1 — Supplementary file1 (PDF 937 KB) [file 13280_2025_2336_MOESM1_ESM.pdf]

Ambio

THIS SUPPLEMENTARY INFORMATION HAS NOT BEEN PEER REVIEWED.

TITLE

Systemic approach to green infrastructure and nature-based solutions uptake: Insights from the Polish cities

AUTHORS AND AFFILIATIONS

Iwona Zwierzchowska[1*], Małgorzata Stępniewska[1], Grzegorz Wolszczak[2]

[1]Department of Integrated Geography, Faculty of Human Geography and Planning, Adam Mickiewicz University in Poznań

[2]Department of Urban, Land, Disaster risk management, World Bank, Vienna, Austria

*Corresponding author

AUTHORS' DETAILS

Iwona Zwierzchowska*
Position: Assistant Professor
Full address: Department of Integrated Geography, Faculty of Human Geography and Planning, Adam Mickiewicz University in Poznań, ul. B. Krygowskiego 10 61-680 Poznań, Poland
Tel. +48618296237
Email: iwona.zwierzchowska@amu.edu.pl

Małgorzata Stępniewska
Position: Associate Professor
Email: malgorzata.stepniewska@amu.edu.pl

Grzegorz Wolszczak
Position: Urban Development Specialist
Email: gwolszczak@worldbank.org

Appendix 1. Framework of Cities' Partnership Initiative - Green Network activities

Date	Location	World Bank expert support: scientific concepts, operational frameworks & tools	Main scope of group work & activities
28.03.2022	Online	• Framing identification of common Green Network themes and shared interest based on participants applications and previously sent questionnaire<br>• Identifying cities interest within specific themes<br>• Identifying potential common areas of work<br>• Facilitating the meeting	• Discussing initial view on Green Network themes, city interest within themes and areas of work<br>• Sharing preliminary ideas for MAPs and their potential benefits
10 -12.05.2022	Lublin	• Introducing the GI/NBS concepts and the methodology framework (inspired by existing frameworks e.g. UrbanByNature, Connecting Nature Framework, PIM, 2018)<br>• Introduction to diagnosis of the city's potential in the context of GI and NBS, including preparation of templates for mapping GI/NBS assets and existing actions, solutions, instruments, and tools applied in the protection, improvement or creation of GI/NBS<br>• Introduction to diagnosis of barriers and constraints to the protection, planning and implementation of GI/NBS, including preparation of templates for mapping organizational, legal, financial and substantive barriers by practitioners<br>• Setting the context for identification of challenges and prioritization of needs for GI/NBS<br>• Leading the workshop work and facilitating group discussion	**Workshop activity:**<br>• City representatives specify and assessed existing GI and shared good practices already applied in practice<br>• City representatives identified and grouped barriers in their activities towards GI/NBS development<br>• City representatives list and organize urban challenges<br>• Open discussion on identified barriers<br>**Sharing knowledge and experience:**<br>• Presentation of cities' approaches to GI & NBS of Lublin, Słupsk and Uniejów<br>• Field visit – approaches to urban green in Lublin
14.07.2022	Online	• Proposed MAPs structure:<br>GI & NBS diagnosis; key challenges and needs; integration MAPs scope and objective with existing policies and plans	• Open Discussion – identification of root causes and contextual factors behind challenges related to GI and NBS; analysis of local, regional, and systemic conditions influencing the emergence of these challenges; use of

		• Presentation of Green Action Plan for Poznań developed under the Health & Greenspace project (URBACT III) as an example of Action Plan	data, evidence, and stakeholder input to support the diagnosis and understanding of GI and NBS-related issues
17 – 19.08.2022	Racibórz, Ruda Śląska, Żory	• Providing guidance for further MAP development rocess, including:<br>• introduction to ecosystem services (ESs) concept and definition (Potschin--Young at al., 2018), ESs classification (Haines-Young and Potschin 2018)<br>• introduction to co-creation canvas concept (Trimi and Berbegal-Mirabent J., 2012; Sparviero, 2019)<br>• Introduction to quintuple helix model (Carayannis, at al., 2012; Carayannis, at al., 2022)<br>• Leading the workshop work and facilitating group discussion	**Workshop activity:**<br>• Facilitated dialogue – MAPs progress status<br>• Canvas-based co-creation session – expected benefits (environmental, social, economic) of planned actions for different beneficiary groups; key actions and required resources; key partners; management measures for the planned solutions<br>**Sharing knowledge and experience:**<br>• Presentation of cities' approaches to GI & NBS – Racibórz, Ruda Śląska, Żory<br>• Field visits – examples of urban green spaces and reclamation and revitalisation of post-industrial sites in Racibórz, Ruda Śląska, and Żory
03 – 06.10.2022	Łódź	• Proposed MAPs structure: task-objective alignment, responsible units, key resources, and implementation timeframe<br>• Gantt chart as a project planning and management tool (e.g. Wilson 2003; Kerzner 2017)<br>• Preparation for financial aspects assessment (barriers and opportunities)<br>• Introducing the Four Natures Approach – urban wastelands role in the city and redevelopment pathways (Kowarik, 2005; Jakubowski, 2020)<br>• Leading the workshop work and facilitating group discussion	**Workshop activity:**<br>• Facilitated dialogue – MAPs Progress Status<br>• Brainstorming – key issues related to financing GI & NBS: investment costs, maintenance costs, stakeholder-specific costs, and opportunities to reduce costs through technical solutions or co-management approaches<br>**Sharing knowledge and experience:**<br>• Presentation of cities' approaches to GI & NBS - Kostrzyn nad Odrą, Gdańsk, Łódź<br>• Field visit – examples of GI & NBS in Łódź
07 – 09.11.2022	Poznań	• Introduction to monitoring and evaluation of GI & NBS (Connecting Nature, 2020)<br>• Proposed MAPs structure: selection of impact indicators (environmental, social, economic); data sources and acquisition methods; monitoring plan	**Workshop activity:**<br>• Facilitated dialogue – MAPs Progress Status<br>• Self-assessment – expected benefits of GI & NBS implementation (according to the Co-Impact Tool, https://co-impact.app/)

		• Leading the workshop work and facilitating group discussion	• Brainstorming – opportunities and limitations of using impact indicators for monitoring and evaluation of GI & NBS actions<br>**Sharing knowledge and experience:**<br>• Presentation of cities' approaches to GI & NBS – Żyrardów, Międzyrzec Podlaski, Poznań<br>• Field visit – examples of GI & NBS in Poznań
14.12.2022	Online	• Introduction to communicating GI and NBS initiatives – objectives, target groups, forms, and communication channels (e.g. Kabisch et al. 2016; Raymond et. al. 2017; Frantzeskaki 2019)<br>• Proposed MAPs structure: communication plan<br>• Leading the workshop work and facilitating group discussion	• Facilitated dialogue – MAPs Progress Status<br>• Open discussion – communication dilemmas
24 – 26.02.2023	Toruń	• Introducing spatial-environmental indictors in local-level spatial planning as a planning tool supporting GI/NBS implementation (Klosse et al., 2022; Szulczewska et al., 2014; Giedych, 2015; Greater London Authority, 2017)<br>• Proposing an approach to identify NBS & GI integration opportunity in the investment processes<br>• Snapshot of recommendations for the systemic development of GI and NBS derived from the Cities' Partnership Initiative 2021–2023<br>• Guiding group work on improvements in coordinating the investment process using NBS and facilitating focus discussion	**Workshop activity:**<br>• Facilitated dialogue – MAPs Progress Status<br>• Parallel group work – developing a proposal for the integration of GI/NBS in the planning, implementation, and maintenance process for a selected (popular or high-conflict) type of investment and improvements for coordination<br>• Open discussion – challenges in incorporating GI & NBS indicators into local spatial planning documents<br>**Sharing knowledge and experience:**<br>• Presentation of Toruń approaches to GI & NBS and field visit – examples of GI & NBS in Toruń
07.03.2023	Online	• Leading discussion on future initiatives that could build on the Cities' Partnership Initiative 2021–2023<br>• Discussing ideas for pilot projects: Development of a model urban GI management plan; Preparation of a model spatial development plan integrating water management at the local or site-specific scale	• Facilitated dialogue – MAPs Progress Status;<br>• Needs for implementation of MAPs<br>• Open discussion – scope, potential, and limitations of preliminary pilot project concepts

27.03.2023	Warszawa	• Project summary presentation: Overview of activities and results of the CIP Green Network	• Final panel discussion – GI and NBS for advancing climate-resilient and livable cities<br>• Closing roundtable – key takeaways from participants<br>**Sharing knowledge and experience:**<br>• Presentation of Warsaw approaches to GI & NBS and field visit – examples of GI & NBS in Warsaw

References:

Carayannis, E.G., T.D. Barth, and D.F.J. Campbell. 2012. The Quintuple Helix innovation model: global warming as a challenge and driver for innovation. *Journal of Innovation and Entrepreneurship* 1: 2. http://dx.doi.org/10.1186/2192-5372-1-2

Carayannis, E.G., D.F.J. Campbell, and E. Grigoroudis. 2022. Helix Trilogy: the Triple, Quadruple, and Quintuple Innovation Helices from a Theory, Policy, and Practice Set of Perspectives. *Journal of the Knowledge Economy* 13: 2272–2301. https://doi.org/10.1007/s13132-021-00813-x

Connecting Nature, 2020. CO-IMPACT tool. Available at https://connectingnature.eu/co-impact-tool (Accessed 20 December 2024)

Frantzeskaki, N. 2019. Seven lessons for planning nature-based solutions in cities. *Environmental Science & Policy* 93: 101–111. https://doi.org/10.1016/j.envsci.2018.12.033

Giedych, R. 2015. Ecological and spatial indicators as a standard for shaping residential development. In *Osiedle mieszkaniowe w strukturze przyrodniczej miasta*, ed. B. Szulczewska, 46-56. Warszawa: SGGW. (In Polish)

Greater London Authority, 2017. Urban Greening Factor for London. Research Report. The Ecology Consultancy.

Haines-Young, R., and M. Potschin. 2018. Common International Classification of Ecosystem Services (CICES) V5.1 and Guidance on the Application of the Revised Structure. Available at www.cices.eu (Accessed 25 July 2025)

Jakubowski, K. 2020. Succession of nature and functions of urban wasteland. Kraków: Fundacja Dzieci w Naturę. (In Polish).

Kabisch, N., N. Frantzeskaki, S. Pauleit, S. Naumann, M. Davis, M. Artmann, D. Haase, S. Knapp, H. Korn, J. Stadler, K. Zaunberger, and A. Bonn. 2016. Nature-based solutions to climate change mitigation and adaptation in urban areas: perspectives on indicators, knowledge gaps, barriers, and opportunities for action. *Ecology and Society* 21(2): 39. http://dx.doi.org/10.5751/ES-08373-210239

Kerzner, H. 2017. *Project Management: A Systems Approach to Planning, Scheduling, and Controlling*. Hoboken, New Jersey: John Wiley & Sons, Inc.

Klosse, M., D. Meksa, J. Misiak, M. Salwa, and D. Stargęga. 2022. More than just a lawn – a biologically active surface in 3D. Miejska Pracownia Urbanistyczna w Łodzi, Łódź. (In Polish). Available at: https://mpu.lodz.pl/files/mpu/public/PROGRAMY_DZIELNIC/wskaznik_zieleni/wskaznik_zieleni.pdf (Accessed 25 July 2025)

Kowarik, I. 2005. Wild urban woodlands: Towards a conceptual framework. In *Wild Urban Woodlands*, ed. I. Kowarik, and S. Körner, 1–32. New perspectives for urban forestry. Berlin: Springer.

PIM, 2018. Guide to Creating an Urban Action Initiative and Local Partnership within the framework of the Partnership Initiative of Cities – a strategic project of the SRD. Partnerska Inicjatywa Miast, Warszawa. (In Polish)

Potschin-Young, M., B. Burkhard, B. Czúcz, and F. Santos Martín. 2018. Glossary for Ecosystem Service mapping and assessment terminology. Deliverable D1.4 EU Horizon 2020 ESMERALDA Project, Grant agreement No. 642007

Raymond, C.M., N. Frantzeskaki, N. Kabisch, P. Berry, M. Breile, M.R. Nita, D. Geneletti, and C. Calfapietra. 2017. A framework for assessing and implementing the co-benefits of nature-based solutions in urban areas. *Environmental Science & Policy* 77: 15–24. https://doi.org/10.1016/j.envsci.2017.07.008.

Sparviero, S. 2019. The Case for a Socially Oriented Business Model Canvas: The Social Enterprise Model Canvas. *Journal of Social Entrepreneurship* 10(2): 232–251. https://doi.org/10.1080/19420676.2018.1541011

Szulczewska, B., R. Giedych, J. Borowski, M. Kuchcik, P. Sikorski, A. Mazurkiewicz, and T. Stańczyk. 2014. How much green is needed for a vital neighborhood? In search for empirical evidence. *Land Use Policy* 38: 330–345. https://doi.org/10.1016/j.landusepol.2013.11.006.

Trimi, S., and J. Berbegal-Mirabent. 2012. Business Model Innovation in Entrepreneurship. *International Entrepreneurship and Management Journal* 8 (4): 449–465. https://doi.org/10.1007/s11365-012-0234-3.

UrbanByNature. The UrbanByNature Integrated Management Approach. Available at https://urbanbynature.eu/our-methodology (Accessed 25 July 2025)

Wilson, J.M. 2003. Gantt charts: A centenary appreciation. *European Journal of Operational Research* 149(2): 430–437. https://doi.org/10.1016/S0377-2217(02)00769-500769-5).
